# Change Blindness in Adolescents With Attention-Deficit/Hyperactivity Disorder: Use of Eye-Tracking

**DOI:** 10.3389/fpsyt.2022.770921

**Published:** 2022-02-28

**Authors:** Michal Hochhauser, Adi Aran, Ouriel Grynszpan

**Affiliations:** ^1^Department of Occupational Therapy, Ariel University, Ariel, Israel; ^2^Neuropedeatric Unit, Shaare Zedek Medical Center, Jerusalem, Israel; ^3^Laboratoire Interdisciplinaire des Sciences du Numérique, Université Paris-Saclay, Orsay, France

**Keywords:** change blindness, attention-deficit hyperactivity disorder, adolescents, eye tracking, fixations, attention, cognitive load

## Abstract

**Objective:**

This study investigated change detection of central or marginal interest in images using a change-blindness paradigm with eye tracking.

**Method:**

Eighty-four drug-naïve adolescents [44 with attention-deficit/hyperactivity disorder (ADHD)/40 controls with typical development] searched for a change in 36 pairs of original and modified images, with an item of central or marginal interest present or absent, presented in rapid alternation. Collected data were detection rate, response time, and gaze fixation duration, latency, and dispersion data.

**Results:**

Both groups' change-detection times were similar, with no speed–accuracy trade-off. No between-group differences were found in time to first fixation, fixation duration, or scan paths. Both groups performed better for items of central level of interest. The ADHD group demonstrated greater fixation dispersion in scan paths for central- and marginal-interest items.

**Conclusion:**

Results suggest the greater gaze dispersion may lead to greater fatigue in tasks that require longer attention duration.

## Introduction

Attention-based mechanisms play an essential role in development, redirecting cognitive resources to new or salient stimuli, and facilitating information processing and adaptive response (1). Attention-deficit hyperactivity disorder (ADHD) is a neurodevelopmental condition characterized by impaired attention, hyperactivity, and impulsivity (2). Although diagnosed most commonly in childhood, it has been shown to be a lifelong disorder that negatively affects everyday functioning (3). Various studies have shown that in adolescence, the inattentional features are dominant and often manifest as limited attention span, disengagement, or distractibility (4). As children with ADHD progress to adolescence, core symptoms of hyperactivity/impulsivity tend to decrease over time, although inattention tends to persist (5, 6). Studies have shown that deficits in visual processing, vigilance, and inhibition continue into adolescence, at the same time, showing inconsistencies in response times impairments specifically in adolescence (7). Thus, adolescents may differ from children with regard to their neuropsychological profile of cognitive control and attention.

A range of experimental paradigms have been used to study different aspects of change detection. For instance, visual-search tests are perceptual tasks requiring attention and active scanning to detect a feature change in a particular object among other objects [i.e., distractors (8)]. In their review, Mullane and Klein (9) concluded that single-feature search (the target differs from the distracters based on one simple perceptive feature, e.g., shape) is relatively preserved in ADHD participants, but serial search (target and distracters share more than one perceptive feature, e.g., shape and color) produced inconsistent results. Although studies of visual search tasks in ADHD provide useful information on detection rates and accuracy in locating an item in an array, they do not offer an exhaustive understanding of change detection, especially when it comes to detecting transient change. Detection of transient change relies on the extended memory span of focal attention to bind together the elements of complex stimuli.

*Change blindness* is a perceptual phenomenon that occurs when a transient change in a visual stimulus is introduced and the observer does not notice it right away (10). Many individuals experience difficulties due to this phenomenon (11, 12) and surprisingly fail to perceive large changes occurring in scenes (12–14). For example, observers often fail to notice major differences introduced into an image while it flickers off and on again.

Experimental paradigms used to test change blindness involve visual attention in such a way that both perceptual and semantic characteristics of the visual scene combine to create a priority list that determines which items to attend first. According to Rensink et al. (10) and Auvray et al. (15), under normal viewing conditions, transient changes in the visual field are automatically detected by low-level perceptual mechanisms. Therefore, attention is extrinsically attracted to the location where the change occurred. Change blindness paradigms are based on the use of experimental protocols successfully masking the local transients that would normally cause the automatic perception of change.

Rensink (16, 17) and Rensink et al. (10) created experimental conditions based on a flicker paradigm task. In the flicker paradigm, an original and modified image of any size and type are presented in rapid alternation with a blank screen placed between successive images. When the blank screen lasts for more than 80 ms, it masks the local transients responsible for automatic perception of change. When attention is no longer automatically directed where change occurs, observers must rely on their scene memory and deliberate control of attention to detect what may have changed. Observers must scan an image, encoding the scene piece by piece (10). Given the number of potential features and objects in a typical natural scene, many aspects of a scene may not be preserved in memory across views. Under this condition, if changes occur in areas that attract more attention, they tend to be detected more quickly. Conversely, if changes occur in areas that attract attention less, they tend to be detected more slowly (18, 19).

An item in the visual field can attract attention due to its perceptual saliency or because of its relevance for the viewer. Studies have found that, in the general population, changes to high-salience features were fixated sooner and for shorter durations and were detected faster and with higher accuracy than those made to low-salience features (20). When saliency features are equalized between different pairs of images in a change-blindness flicker task, differences in change detection will be guided mostly by the viewer's interest for the objects displayed on the images. Rensink et al. (10) characterized these differences as related to the semantic properties of the objects in the images and showed higher performances in the detection of items of central interest (attracting more attention) compared to those of marginal interest (attracting less attention).

In such experimental tasks, change detection performances rely on prolonged search periods and shifts of focal attention to the change for it to become conscious. Focal attention to change is not sufficient for conscious detection of change in change blindness flicker tasks: attention can be directed to changes—that is, changes can be stared at “blankly,” without the change being perceived. A further consolidation process supported by working memory seems to be required for conscious detection (21, 22). Interestingly, Martinussen et al.'s (23) meta-analysis showed spatial working memory in children with ADHD compared to controls.

Few studies have investigated change blindness in individuals with ADHD and, as far as known to the authors, none with adolescents. Cohen (24) found that children with ADHD were faster than typically developing children in specifically detecting marginal changes. The authors presumed that that the children with ADHD possibly had not utilized the stereotyped, repetitive scan path that typifies central interest changes in typically developing children. Instead, they may have used a less systematic, more disorganized scan path, which paradoxically happened to serve them in identifying marginal interest changes in the flicker task. In contrast, Maccari et al. (25) found that children with ADHD performed more slowly and less accurately than controls in detecting marginal changes. Cohen and Shapiro (26) found no differences in change-detection times between adults with and without ADHD, but those with ADHD had more commission errors compared to controls, indicating a speed–accuracy performance tradeoff. These inconsistencies may have stemmed from inclusion criteria, treatment differences between groups, or methodological differences.

Türkan et al. (27) used eye tracking in a change-blindness study and found that children with ADHD made shorter fixations on the changed area than did typically developing children. The change-detection performances of children with ADHD were also less accurate compared to TD children. These findings aligned with known difficulties in ADHD with sustaining the attention necessary to encode the scene properties and goal-oriented behavior.

However, different experimental paradigms using eye tracking can yield contrasting outcomes. For instance, Karatekin and Asarnow (28) used a task in which children were asked to explore static pictures to answer specific questions. They found no difference between participants with ADHD and typical development in the time spent viewing relevant and irrelevant regions, fixation duration (an estimate of processing rate), or distance between fixations. In contrast, in a different study, students with ADHD had more fixations, which were also significantly shorter than were the controls' fixations (29). Furthermore, an additional study found that although the ADHD and typical development groups did not differ in fixation duration, visual scanning of children with ADHD was discontinuous, uncoordinated, and chaotic compared with the controls (30).

In summary, few studies have used a change-blindness paradigm to investigate visual attention in people with ADHD. These studies reported contradicting results regarding change-detection performance of people with ADHD. They investigated change blindness in children (24, 25, 27), and one investigated adults (26), whereas the adolescent population has not yet been studied. Only one change blindness study used an eye-tracker device (27).

Thus, the aim of this study was two-fold. The first was to compare the change-detection performance of adolescents with ADHD to that of adolescents with typical development, using a change-blindness paradigm. We compared change detection time and error rates in items of central and marginal interest. An additional goal was to refine the investigation of the attentional process by examining gaze patterns using eye tracking. We compared time to first fixation (TFF) on the changed item (i.e., first-fixation latency), and total fixation duration (TFD) on the changed item (i.e., total time spent fixating on the changed item). We also compared scan paths and gaze dispersion.

We hypothesized that ADHD participants would have longer change-detection times, particularly in detecting marginal interest changes because they require greater attentional control, whereas central interest changes semantically pop out from the picture (10). Alternatively, we expected that ADHD participants would have a higher error rate because a speed–accuracy tradeoff usually occurs.

We also expected that limited attentional resources would cause participants with ADHD to have longer gaze TFD on the marginal interest changed items compared to the group with typical development. We assumed that, because detecting marginal change requires an item-by-item scan of the entire image using comparison strategies and working memory, it gives rise to longer identification times if processing is inefficient or speed is slowed. We hypothesized that first-fixation latency (TFF) would be longer for marginal interest changed items for the same reasons. These in turn, we speculated, might cause the ADHD group to exhibit longer scan paths and greater gaze dispersion because they might need to often backtrack to detect the change.

## Methods

### Participants

The sample size was calculated by using G^*^Power for detecting moderate effects with a statistical power of 95%, with alpha at 0.05 (31). The initial sample included 89 adolescents, with an age range of 12–19 years, without evident motor disturbances or intellectual or neurological impairments. Five participants (three with ADHD, two with typical development) had insufficient data due to technical difficulties. Consequently, the final sample consisted of 84 adolescents.

The ADHD group comprised 44 participants (15 girls, 34.1%; 29 boys, 65.9%); the group with typical development (TD group) comprised 40 participants (18 girls, 45.0%; 22 boys, 55.0%). The groups did not differ with respect to age (Table 1). Adolescents who were diagnosed with ADHD were recruited from the Pediatric Neurology Unit of the Shaare Zedek Medical Center. The diagnosis of ADHD was made by an experienced clinician based on criteria of the *Diagnostic and Statistical Manual of Mental Disorders* (2). It included the Disruptive Behavior Disorders Rating Scale (DBDRS) and a structured diagnostic interview with the parents. Adolescents were included if they scored outside the normal range on the inattention or hyperactivity/impulsivity subscale of the DBDRS, wherein six or more items must be endorsed as “pretty much” or “very much” to meet criteria. Inclusion criteria for both the ADHD and TD groups included absence of chronic medications and attending a regular classroom setting.

**Table 1:** Participant characteristics in change blindness experiment.

**Characteristic**	**TD (*N* = 40)** ***Mean ±SD***	**ADHD (*N* = 44)** ***Mean ±SD***	***p***
Age	14.6 ± 2.13	14.6 ± 2.06	0.89
Gender	*Male:22; female:18*	*Male:29; female:15*	0.37
	1.45 ± 0.5	1.34 ± 0.48	

*TD, group with typical development; ADHD, group with attention-deficit/hyperactivity disorder*.

The ADHD group included 15 children who met the criteria for the ADHD/C subtype (exhibit both inattentiveness and hyperactivity/impulsiveness symptoms), 14 who met the criteria for ADHD/A (show predominantly inattentive symptoms), and 15 who met the criteria for ADHD/B (show prevalently hyperactivity/impulsivity symptoms).

Adolescents in the comparison (TD) group were selected from a local school and attended a regular classroom setting but were excluded from the study if they had above-cutoff scores on the DBDRS. The study was approved by the [blinded] University's Ethical Review Board and by the Helsinki Committee of the Hospital. A written informed assent and consent was obtained from the participants and their parents signed an informed consent.

### Materials and Apparatus

The experimental stimuli consisted of 36 pairs of real-world scene images, 18 of which were adopted from Rensink et al. (10) and the other 18 from selected from Hochhauser et al. (32). Two additional image pairs from the same sources were used for practice trials. Each picture measured 1,008 × 720 pixels. Each pair of images were identical, apart from a single difference in the presence or absence of a particular object or area.

Following the method indicated by Rensink et al. (10), the pairs of images were divided into two equal groups where changes were either of high-level interest (*central*) or of low-level interest (*marginal*). The images were matched on psychometric properties, location of change, size of change, conspicuity of change, and intensity (33). The images had been validated for use for the change-blindness paradigm in previous studies (10, 32).

The images were displayed on a 17-in screen in a “flicker” paradigm from a viewing distance of about 50 cm. Responses were collected *via* the computer mouse. The stimuli were presented using a Tobii X2-60 eye tracker, which is non-invasive and allows for free, unconstrained movement of the head and body (www.tobii.com). For each image, we analyzed gaze data on an AOI that circumscribed the changing item with an 0.5-visual degree margin (5 mm) to account for the eye tracker's accuracy error. We computed TFD and TFF based on fixations that fell in this AOI.

### Procedure

**Figure 1:**
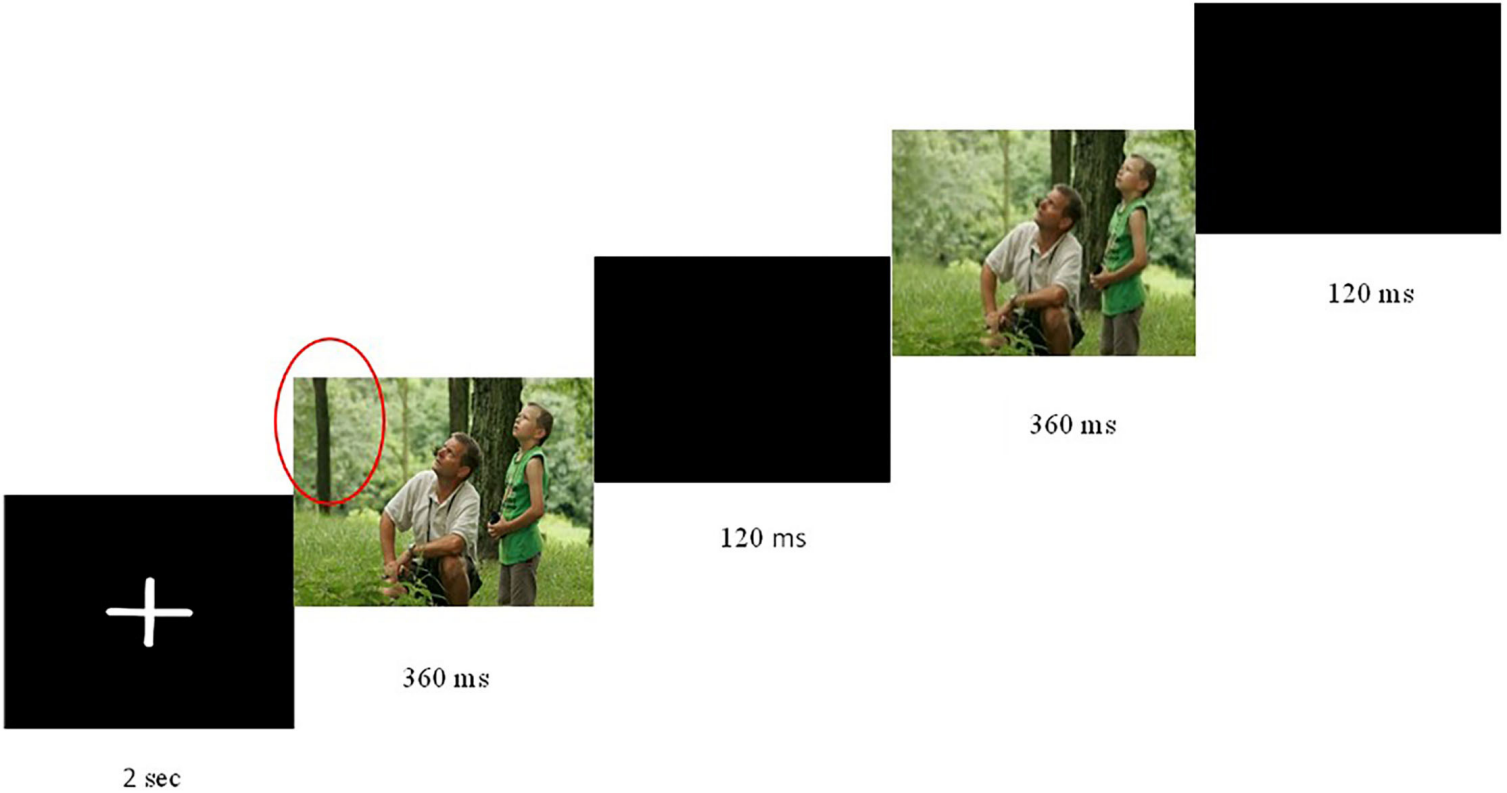
Flicker paradigm used to test change blindness. A pair of images was presented, each for 360 ms, with an intervening blank screen of 120 ms. Cycle repeated until participants responded or 45 s elapsed. Image accessed at Wikimedia Commons, the free media repository.

The images were presented in counterbalanced trials in two test blocks with a 1–2 min rest period in between. Each trial started with a fixation cross (apparent size in visual angles: 1 × 1°) appearing for 1 s. It was followed by a briefly displayed (360 ms) scene image, followed by a gray blank scene displayed for 120 ms, followed by another image displayed for 360 ms, and a repetition of this sequence (Figure 1). The sequence alternated between a scene and its modified version, repeating itself until the participant clicked the mouse or until 45 s had elapsed. This response was based on the task instructions, which specified that the participants had to indicate as quickly as possible, but without guessing, what the change in the scene was. The response time measure, recorded in milliseconds, was the time at which the mouse click occurred. Following the mouse click, the participant had to verbally describe the change to the experimenter. For the trial to be classified as a correct response, the participant had to verbally identify and locate the changing item. Trials in which the participant made a mouse click response but did not correctly identify the item that changed in the scene were scored as an error. Two practice trials were conducted before starting the main experiment. During the actual experiment, no feedback was provided.

Participants were tested individually in a silent room. Before they began, they received a brief overview of the experiment and were instructed to click on the mouse as soon as they detected that one object appeared and disappeared, and then to verbally describe the change. Participants were told that a change would occur on every trial and were encouraged to keep searching for differences and not give up before the end of the trial. A 9-point calibration of the eye tracker was applied before each experiment.

### Data Analysis

A group (ADHD, Control) × level of interest (central, marginal) repeated measures analysis of variance (ANOVA) was carried out for change-detection times (i.e., time to first mouse click) and eye-tracking metrics. The ANOVA for percentage of errors included an additional factor for the type of error (omission or commission; that is, missed items or incorrect detections). Change-detection times and eye-tracking metrics in trials in which participants did not detect the change were not included in the analyses. Eye-tracking metrics included the TFF (the amount of time that it took the respondent to first gaze at the changing item), TFD on the changing item, mean scan path (average of the distance between every two sequential points), total scan path (total distance between successive fixations), and gaze dispersion. Gaze dispersion was computed by identifying the smallest convex area enclosing all the fixation points. A convex hull was computed for each trial of every participant with Matlab^®^. The areas of the convex hulls were then averaged over the trials of each participant in each condition to provide a metric for dispersion. Data was analyzed by using IBM SPSS Statistics for Windows, Version 23.0. When appropriate, *post-hoc t*-tests were performed using the Bonferroni adjustment.

## Results

### Percentage of Errors

There were no errors for the central level of interest in either the ADHD or TD group; therefore, data analysis was applied only for the marginal level of interest. There was a main effect for type of error, *F*_(1, 82)_ = 7.03, *p* ≤ 0.01, η^2^ = 0.01 (ADHD: omission *M* ± SD = 0.004 ± 0.02, commission *M* ± *SD* = 0.002 ± 0.01; TD: omission *M* ± *SD* = 0.01±0.02, commission *M* ± *SD* = 0.00 ± 0.005). However, the percentage of errors did not differ between groups, *F*_(1, 82)_ = 0.83, *p* = 0.36, nor was there an interaction effect between type of error (i.e., omissions/missed items or commissions/incorrect detections) and group, *F*_(1, 82)_ = 2.6, *p* = 0.11.

### Change Detection Times

Table 2 presents the data for change-detection times (time to first mouse click), TFF, TFD, scan paths, and fixation dispersion for central- and marginal-level of interest among the adolescents with ADHD and with TD.

**Table 2:** Change-blindness task: results of repeated measures ANOVA, response times (s), duration (s), scan path (mm), and gaze dispersion by group.

**Change blindness**	**TD (*****N*** **=** **40)** **(M** **±SD)**		**ADHD (*****N*** **=** **44)** **(*****M ±SD*****)**		**Level of interest** ***df*****(1, 82)**		**Group effect** ***df*****(1, 82)**		**Interaction effect** ***df***_**(1, 82)**_	
**Level of interest**	**Central**	**Marginal**	**Central**	**Marginal**	***F***	**η** ^**2**^	***F***	**η** ^**2**^	***F***	**η** ^**2**^
Time to first mouse click	3.00 ± 0.97	8.03 ± 2.43	3.66 ± 1.35	8.25 ± 2.73	363.9^***^	0.82	0.02	–	0.73	–
Time to first fixation	1.07 ± 0.58	4.23 ± 1.91	1.18 ± 0.59	4.24 ± 2.12	236.4^***^	0.75	0.05	–	0.08	–
Total fixation duration	0.88 ± 0.47	1.15 ± 0.66	1.01 ± 0.55	1.30 ± 0.71	31.1^***^	0.28	0.01	–	0.03	–
Scan path	7.23 ± 3.77	6.91 ± 3.48	8.41 ± 5.51	7.45 ± 3.32	6.21^**^	0.07	1	–	1.6	–
Total scan path	18.68 ± 11.37	58.93 ± 32.86	22.51 ± 13.32	63 ± 44.68	122^***^	0.6	0.57	–	0.001	–
Gaze dispersion	0.02 ± 0.01	0.03 ± 0.02	0.03 ± 0.02	0.04 ± 0.02	40.4^***^	0.32	5.72^**^	0.1	0.39	–

*TD, group with typical development; ADHD, group with attention-deficit/hyperactivity disorder; Mean scan path, average distance between fixations; Total scan path, total distance between successive fixations; Gaze dispersion, the smallest convex area enclosing all the fixation points*.

** *p ≤ 0.01;*

*** *p ≤ 0.001*.

**Figure 2:**
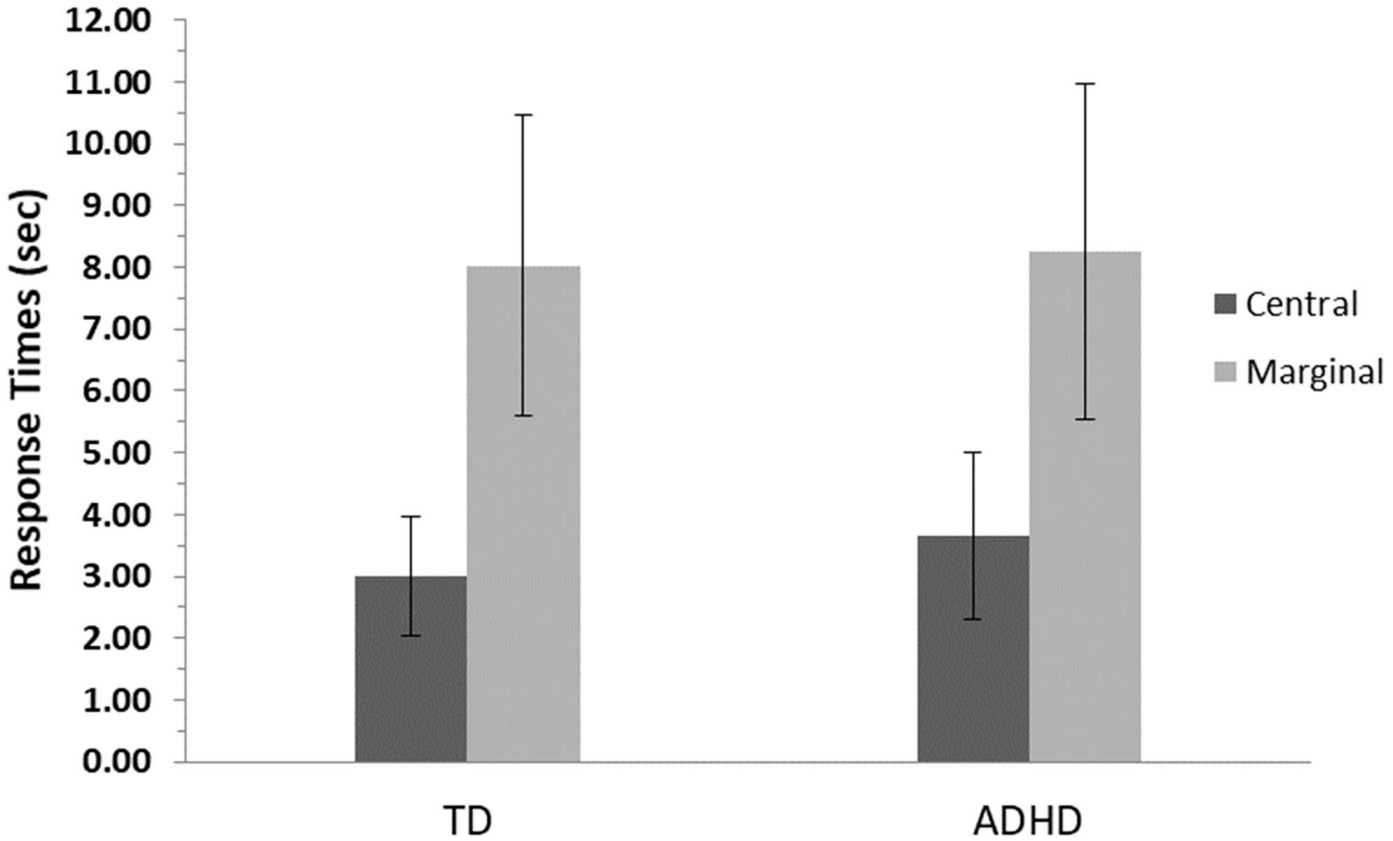
Detection time means and standard errors for central- and marginal-interest changes by group. TD, group with typical development; ADHD, group with attention-deficit/hyperactivity disorder. Detection time measured as time to first mouse click. A significant difference was found between levels of interest (central/marginal) for the two groups (*p* ≤ 0.001); however, no differences were found between groups.

Detection times (time to first mouse click) were longer for marginal items compared with central items, *F*_(1, 82)_ = 363.9, *p* ≤ 0.001, η^2^= 0.82. There was no significant difference between the ADHD and TD groups, *F*_(1, 82)_ = 0.02, *p* = 0.26, and no interaction effect between groups and levels of interest, *F*_(1, 82)_ = 0.73, *p* = 0.84 (Figure 2).

### Changed Items TFF

**Figure 3:**
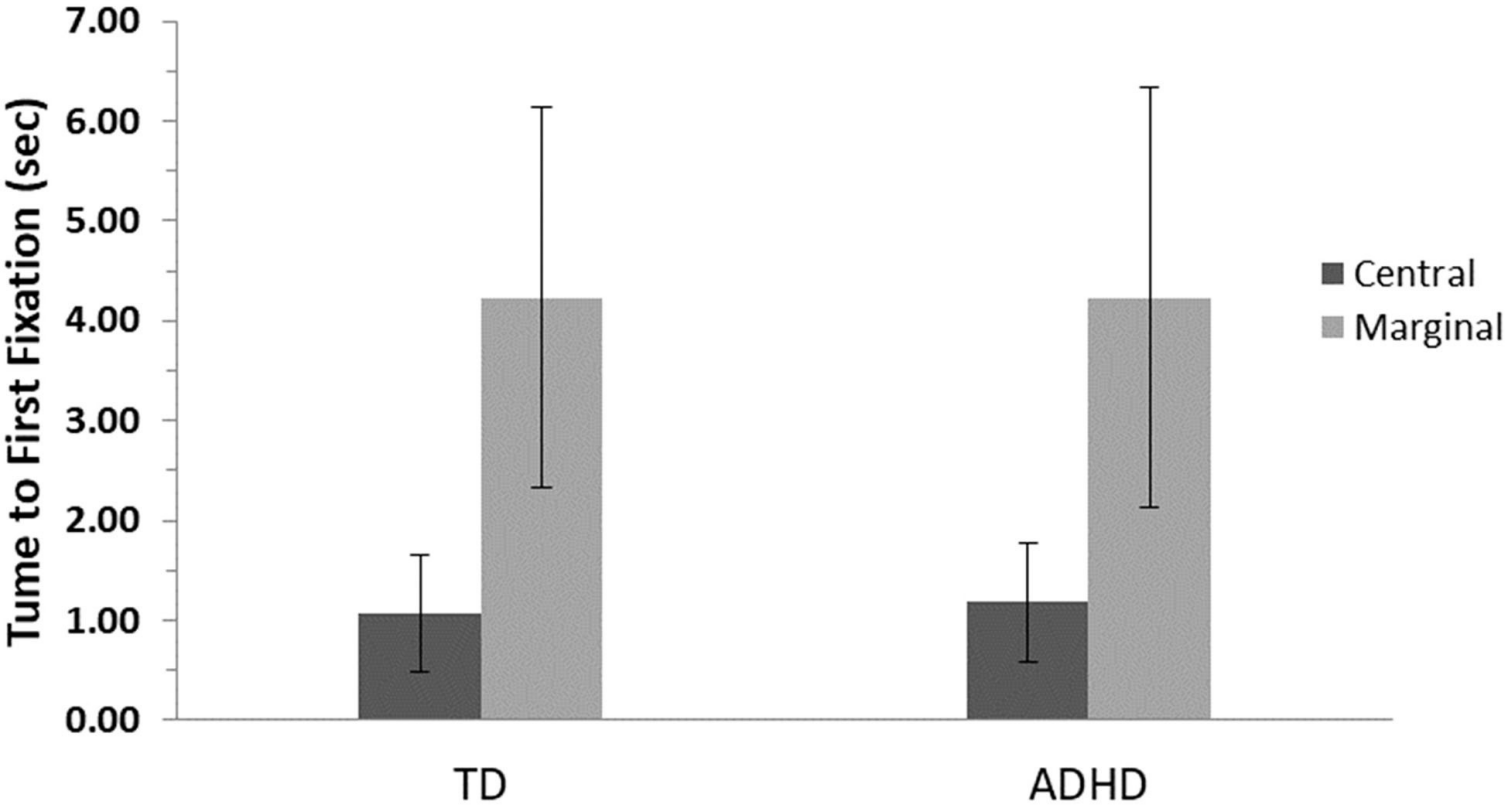
First fixation means and standard errors for central- and marginal-interest changes by group. TD, group with typical development; ADHD, group with attention-deficit/hyperactivity disorder. A significant difference was found between levels of interest (central/marginal) for the two groups *(p* ≤ 0.001); however, no differences were found between groups.

The results revealed a main effect for level of interest, *F*_(1, 82)_ = 236.4, *p* ≤ 0.001, η^2^ = 0.75, showing that both groups were slower to look at the marginal items, but no significant difference between groups, *F*_(1, 82)_ = 0.05, *p* = 0.83, and no interaction effects, *F*_(1, 82)_ = 0.08, *p* = 0.79, were found (Figure 3).

### Changed Items TFD

**Figure 4:**
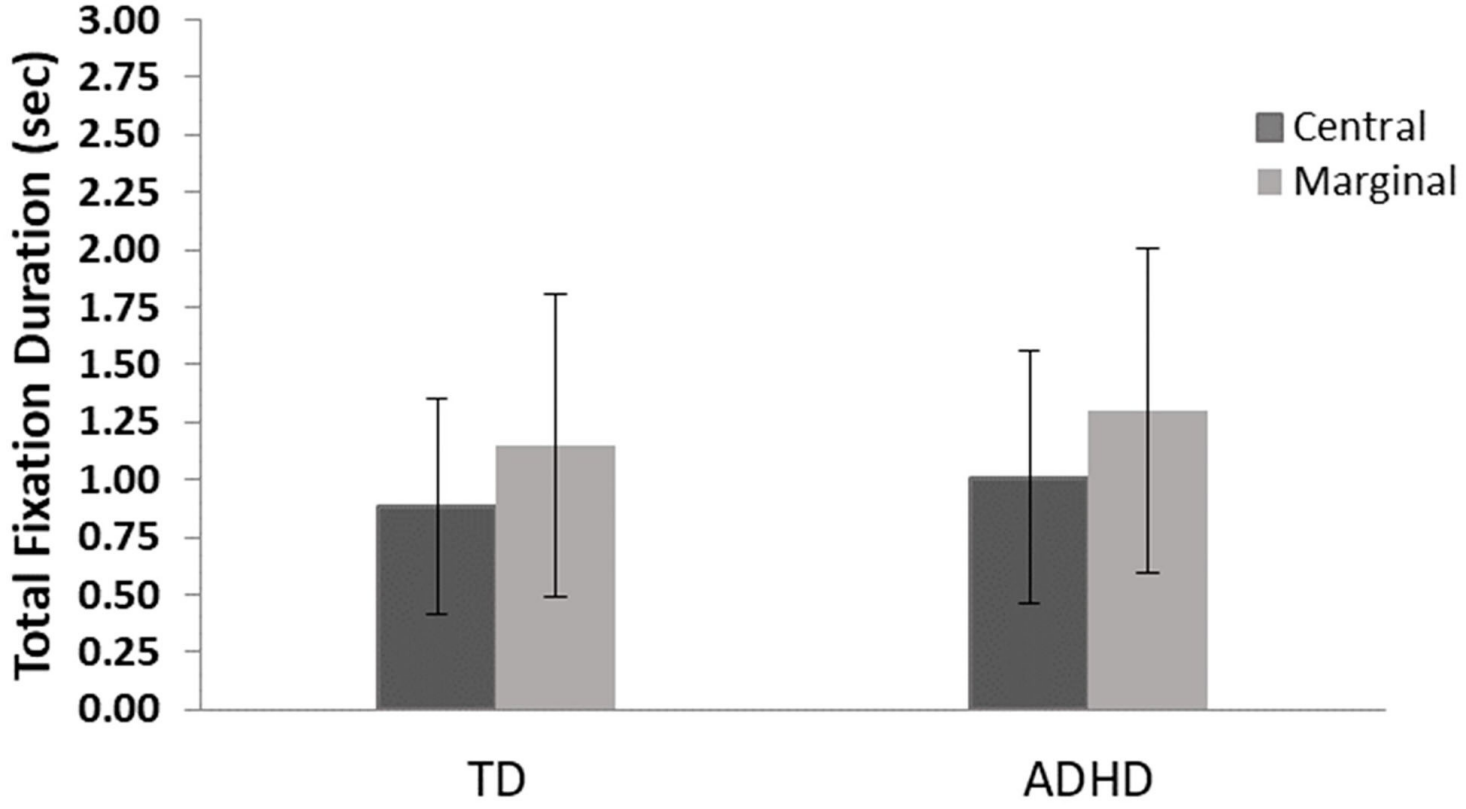
Total fixation duration means and standard errors for central- and marginal-interest changes by group. TD, group with typical development; ADHD, group with attention-deficit/hyperactivity disorder. A significant difference was found between levels of interest (central/marginal) for the two groups *(p* ≤ 0.001); however, no differences were found between groups.

The results revealed a main effect for level of interest, *F*_(1, 82)_ = 31.1, *p* ≤ 0.001, η^2^ = 0.28, showing that both groups looked longer at the marginal items, but no significant difference between groups, *F*_(1, 82)_ = 0.01, *p* = 0.72, and no interaction effects, *F*_(1, 82)_ = 0.03, *p* = 0.57 were found (Figure 4).

### Scan Paths

Mean scan path (average distance between sequential points) findings for the ADHD group (Central: *M* = 8.41, *SD* = 5.51; Marginal: *M* = 7.45, *SD* = 3.32) and controls (Central: *M* = 7.23, *SD* = 3.77; Marginal: *M* = 6.91, *SD* = 3.48) revealed a main effect for levels of interest, *F*_(1, 82)_ = 6.21, *p* ≤ 0.01, η^2^= 0.07, but no interaction effect, *F*_(1, 82)_ = 1.6, *p* = 0.21, or between-group effect *F*_(1, 82)_ = 1, *p* = 0.32.

Total scan path (total distance between successive fixations) findings for the ADHD group (Central: *M* = 22.51, *SD* = 13.32; Marginal: *M* = 63, *SD* = 44.68) and controls (Central: *M* = 18.68, *SD* = 11.37; Marginal; *M* = 58.93, *SD* = 32.86) revealed a main effect for levels of interest, *F*_(1, 82)_ = 122, *p* ≤ 0.001, η^2^ = 0.6, but no interaction effect, *F*_(1, 82)_ = 0.001, *p* = 0.97, or between-group effect, *F*_(1, 82)_ = 0.57, *p* = 0.45.

### Gaze Dispersion

**Figure 5:**
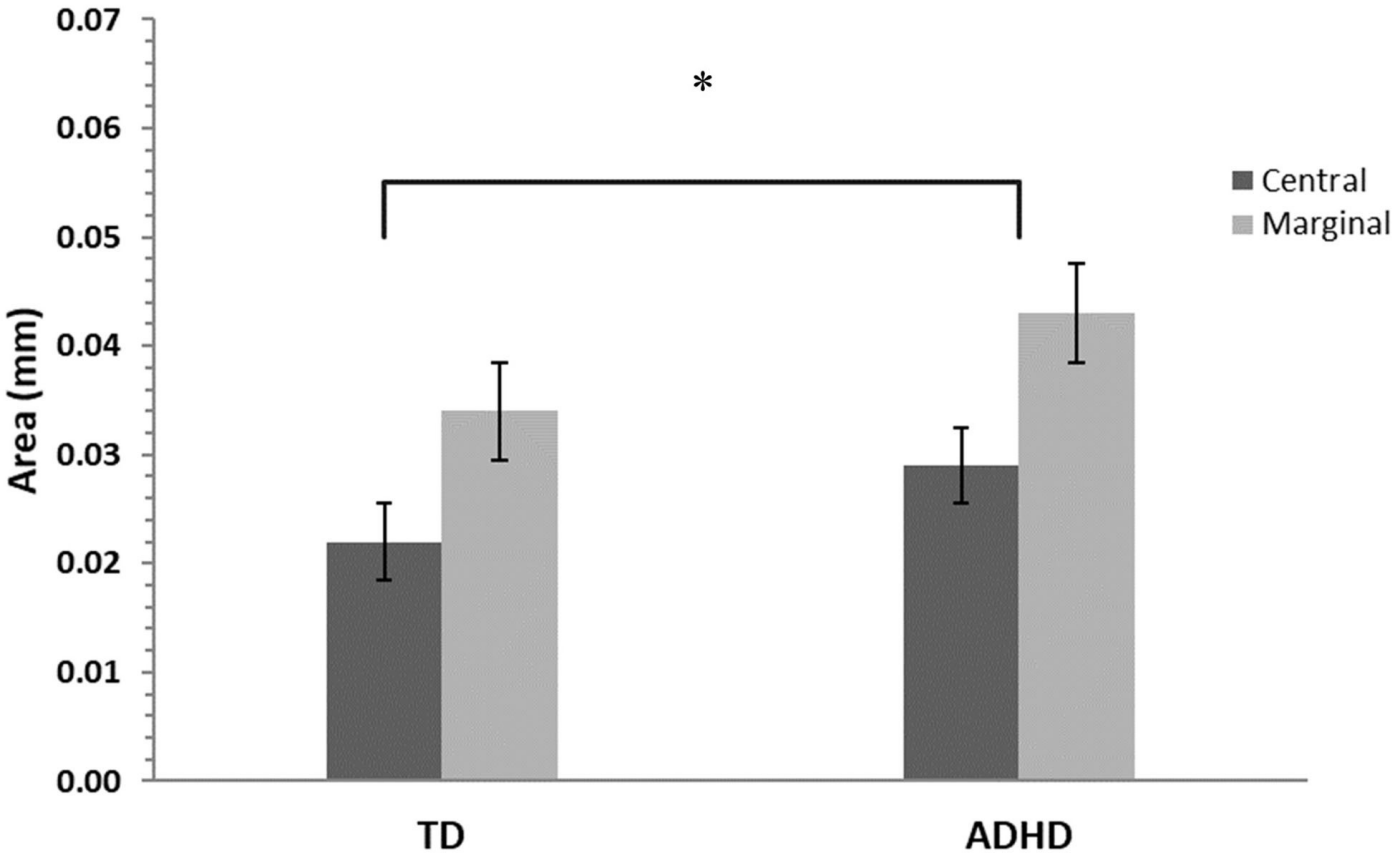
Gaze dispersion means and standard errors for central- and marginal-interest changes by group. TD, group with typical development; ADHD, group with attention- deficit/hyperactivity disorder. A significant difference was found between groups (*p* ≤ 0.01) and between levels of interest (central/marginal) for the two groups *(p* ≤ 0.001), however there was no interaction effect. The asterisk is to show significance.

Gaze dispersion (area of the convex hull of fixation points) analysis revealed a main effect for levels of interest, *F*_(1, 42)_ = 40.4, *p* ≤ 0.001, η^2^ = 0.32, and a main effect for groups, *F*_(1, 42)_ = 5.72, *p* ≤ 0.01, η^2^ = 0.1, with no interaction effect for these two factors, *F*_(1, 42)_ = 0.39, *p* = 0.54 (Figure 5).

## Discussion

The purpose of this study was to compare the visual attention of adolescents with and without ADHD. We used a change-blindness task on an age group that has not been studied before, that is, adolescence. Contrary to our hypothesis and a previous study among children (25), the adolescent ADHD group performed similar to the controls in the number of errors, response times, and TFF, as well as TFD on the changing item and measures of scan paths. Moreover, similar to the controls, the ADHD group demonstrated greater response times, greater time to first fixation, longer fixation durations, and longer scan paths when detecting marginal changes compared to central changes. Nonetheless, the results showed a significant difference between the ADHD group and controls in their gaze fixation dispersion. The adolescents with ADHD demonstrated larger spatial coverage than did their typically developing peers.

The lack of difference between the ADHD and TD groups on performance metrics such as the detection rate and detection times can be interpreted in light of the perceptual cognitive load theory (34). According to theory, a major determinant of the ability to focus attention while eluding distraction is whether the task being performed involves a sufficiently high “perceptual load.” *Perceptual load* has been operationally defined as either the quantity of stimuli entailing perceptual processing or the complexity of perceptual judgments (35). When the task processing involves high load (e.g., requiring complex perceptual judgments or searching among many items), perception of distractors is reduced or even eliminated. Moreover, although beyond the main scope of this paper, cognitive load has found to influence oculomotor behavior (36, 37).

In the current study, the change-blindness task necessitated complex perceptual judgement. For example, the marginal condition required inhibition of distracting features that were semantically relevant to the scene displayed, although they were irrelevant to the required task of detecting change. The analysis of results did not yield evidence that change-detection times were longer in participants with ADHD compared to those with typical development. The ADHD group did not show different speed–accuracy tradeoffs that could be a sign of them being tired or having difficulties concentrating. Hence, the “perceptual cognitive load” required by the change-blindness task may have helped those with ADHD to focus. This explanation is in line with a study that tested the perceptual cognitive load theory in individuals with ADHD (38) and whose results indicated that conditions of increased perceptual cognitive load facilitated task engagement in people with ADHD with reduced distraction. We therefore suggest that, although it may seem counterintuitive, performing a challenging task with a high cognitive load might have in fact facilitated the performances of adolescents with ADHD in the present study.

Time to first fixation and TFD on the changed items followed the same trend as percentage of errors and detection times—that is, there were no differences between participants with ADHD and the TD controls. These results contrast with the results of Türkan et al. (27), who found shorter fixations in children with ADHD and less accurate detection performances. This could suggest that, as they reach adolescence, individuals with ADHD may be able to perform as well as their typically developing peers in change-blindness tasks.

Nonetheless, one can deliberate that in the wake of the current knowledge of executive functioning difficulties, such as working memory, in people with ADHD (39), the task should have challenged them. As mentioned earlier, working memory plays a central role in change-blindness tasks (21, 22). A study by Yeh et al. (40) revealed that young adults with better working-memory capacities gazed at the targets more frequently and for longer times than did participants with lower working-memory capacities. It was therefore reasonable to expect differences in eye-tracking measures in the present study due to the weaker working-memory performances reported with ADHD (23).

A feasible argument for the ability of the participants in the current study to overcome working-memory difficulties is compensatory attentional processes, such as stimulus-driven attention, as opposed to goal-directed attention. Working-memory deficits might not affect attentional control adversely in individuals with ADHD when the task design or stimuli provide extrinsic reinforcement of the task set and rules. For instance, Burgess et al. (41) administered the Color-Word Stroop task to participants with ADHD and found they had poorer performances only for the congruent condition; that is, when color-words were written in the same ink color as the word. Those authors suggested that the inherent conflict between the ink color and the word provided a subtle reminder of the task sets and rules.

Burgess et al.'s (41) results suggest that intrinsic working-memory difficulties affect attentional control less when task stimuli support task-set maintenance. The flicker used to implement the change-blindness paradigm in the present experiment may have acted as a reminder of the task sets by generating a regular stimulus-driven signal of the change between the two versions of the displayed image. This aligns with Corbetta and Shulman (1), who maintained that two networks of brain areas are involved in controlling attention: The first one relies on cognitive information to direct attention to relevant objects in a visual scene; the second is associated with attention control driven by stimulus properties rather than cognitive processes (e.g., “bottom-up” control of attention, which explains why people are drawn to “oddball” stimuli that are very different from the background). As such, the flicker-based change-blindness paradigm used in the current study differs from more conventional visual-search tasks in that it provides a stimulus-driven reminder of the task sets throughout the duration of the task.

Differences were nevertheless found when analyzing gaze dispersion. Those differences did not appear in mean and total scan paths measures, probably because such metrics were too coarse. They did appear, however, when considering the convex hulls of fixation points. It appears that the participants with ADHD tended to survey a larger portion of the image. The increase in gaze fixation area may reflect a greater proclivity to attend to a new stimulus, even when asked to goal orient on detecting a change, and a relatively attenuated propensity to continue processing the current stimulus, creating fixation scatter.

It is further possible that “fixation scatter” may reflect an imbalance between the bottom-up stimulus-driven and the top-down attentional-control mechanisms that govern visual attention (1). These results showing greater gaze dispersion are consistent with the findings of Jayawardena et al. (42), who revealed that adults with ADHD do not visually scan stimulus items using a path similar to adults without ADHD. Krejtz et al. (43) also indicated that although adults with ADHD had fixations to salient visual cues similar to adults without ADHD, they demonstrated less structured and more chaotic scan patterns. Mohammadhasani et al. (30) found that, compared to children with typical development, children with ADHD did not follow a typical scan path; instead, their visual scanning was discontinuous, uncoordinated, and chaotic. In Munoz et al.'s (44) study investigating the control of visual fixation in a task requiring prolonged fixation, ADHD participants generated more intrusive saccades during periods when they were required to maintain steady fixation. The authors proposed that ADHD participants have reduced ability to suppress unwanted saccades and control their fixation behavior voluntarily, which is consistent with the fronto-striatal pathophysiology linked to difficulty in inhibition.

This is the first study to our knowledge that used change blindness to assess visual attention in adolescents with ADHD. Two previous studies (25, 27) showed that children without attention deficits perform better in change-detection tasks than do those with ADHD, whereas Cohen and Shapiro (26) showed there were no differences in adults. The present study with adolescents showed no differences on performance metrics (percentage of errors and detection times), TFF, and TFD on the changing items but did reveal an increased dispersion of gaze. Of particular relevance is the fact that the stimuli were presented in a flicker paradigm, which perhaps biased the participants (i.e., attracted and maintained attention). However, previous ADHD studies on change blindness relied on the same paradigm. Future studies can address this limitation by implementing change-blindness experiments without a flicker as, for instance, those based on continuity errors in film cuts (45). Additional limitations were that the participants were not formally tested for IQ; thus, the ADHD and control group were not matched on IQ scores. However, participants' academic levels were queried, and the groups were matched to them.

An additional issue for further investigation is that of ecological validity; that is, the extent to which the stimuli and protocol approximate the real-life situation of adolescents with ADHD. This study may shed light on the visual-attention patterns adolescents with ADHD exhibit when required to attend to images portraying various real-life contexts. However, we strongly recommend investigating whether the conclusions hold when associated with everyday tasks that require “functional attention.”

## Conclusions

This study attempted to gain insight on the attentional performances of adolescents with ADHD, using a change-blindness paradigm with naturalistic images. The use of eye tracking enabled analyzing gaze patterns, such as TFF and TFD on the changing item, as well as the scan paths and fixation dispersion. Adolescents with ADHD detected changes with similar accuracy and speed compared to controls but with gaze dispersed across larger areas. Our results with regards to accuracy should not be taken as conclusive because there possibly was a ceiling effect related to the difficulty of the tasks, and that more difficult tasks would result in some more subtle between-group differences. The greater gaze dispersion in the ADHD group sheds light on the distinctive attentional mode of people with ADHD, suggesting less structured gaze patterns and a lack of inhibition of intrusive saccades. Future directions should investigate whether this gazing behavior is beneficial over time, or if it causes fatigue or lower efficiency when completing longer tasks. Furthermore, this study substantiates the potential assets of eye tracking as a comprehensive tool for assessing attentional deficits in those who are suspected of having ADHD.

## Data Availability Statement

The raw data supporting the conclusions of this article will be made available by the authors, without undue reservation.

## Ethics Statement

The studies involving human participants were reviewed and approved by Helsinki Committee Shaare Zedek Medical Center. Written informed consent to participate in this study was provided by the participants' legal guardian/next of kin.

## Author Contributions

MH conceptualized, designed and performed experiments, analyzed data, and co-wrote the paper. AA provided Helsinki approval management and medical guidance. OG analyzed data and co-wrote the paper. All authors contributed to the article and approved the submitted version.

## Funding

Open access publication fees received from Ariel University.

## Conflict of Interest

The authors declare that the research was conducted in the absence of any commercial or financial relationships that could be construed as a potential conflict of interest.

## Publisher's Note

All claims expressed in this article are solely those of the authors and do not necessarily represent those of their affiliated organizations, or those of the publisher, the editors and the reviewers. Any product that may be evaluated in this article, or claim that may be made by its manufacturer, is not guaranteed or endorsed by the publisher.

## References

[1] Corbetta M Shulman GL. Control of goal-directed and stimulus-driven attention in the brain. Nat Rev Neurosci. (2002) 3:201–15. 10.1038/nrn755 11994752

[2] American Psychiatric Association. Diagnostic and Statistical Manual of Mental Health Disorders (5th ed.). Arlington, VA (2019).

[3] Barkley RA (editor). Attention-Deficit Hyperactivity Disorder: A Handbook for Diagnosis and Treatment (4th ed.). New York, NY: Guilford Press (2015).

[4] Wehmeier PM Schacht A Barkley RA. Social and emotional impairment in children and adolescents with ADHD and the impact on quality of life. J Adolesc Health. (2010) 46:209–17. 10.1016/j.jadohealth.2009.09.009 20159496

[5] Francx W Zwiers MP Mennes M Oosterlaan J Heslenfeld D Hoekstra PJ. White matter microstructure and developmental improvement of hyperactive/impulsive symptoms in attention-deficit/hyperactivity disorder. J Child Psychol Psychiatry. (2015) 56:1289–97. 10.1111/jcpp.12379 25581343 PMC4499023

[6] Willcutt EG Nigg JT Pennington BF Solanto MV Rohde LA Tannock R. Validity of DSM-IV attention deficit/hyperactivity disorder symptom dimensions and subtypes. J Abnorm Psychol. (2012) 121:991. 10.1037/a0027347 22612200 PMC3622557

[7] Franke B Michelini G Asherson P Banaschewski T Bilbow A Buitelaar JK. Live fast, die young? A review on the developmental trajectories of ADHD across the lifespan. Euro Neuropsychopharmacol. (2018) 28:1059–88. 10.1016/j.euroneuro.2018.08.001 30195575 PMC6379245

[8] Treisman AM Gelade G. A feature-integration theory of attention. Cogn Psychol. (1980) 12:97–136. 10.1016/0010-0285(80)90005-5 7351125

[9] Mullane JC Klein RM. Literature review: visual search by children with and without ADHD. J Attent Disord. (2008) 12:44–53. 10.1177/1087054707305116 17712165

[10] Rensink RA O'Regan JK Clark JJ. To see or not to see: the need for attention to perceive changes in scenes. Psychol Sci. (1997) 8:368–73. 10.1111/j.1467-9280.1997.tb00427.x

[11] Simons DJ Ambinder MS. Change blindness: theory and consequences. Curr Dir Psychol Sci. (2005) 14:44–8. 10.1111/j.0963-7214.2005.00332.x

[12] Simons DJ Rensink RA. Change blindness: past, present, and future. Trends Cogn Sci. (2005) 9:16–20. 10.1016/j.tics.2004.11.006 15639436

[13] Simons DJ. Current approaches to change blindness. Vis cogn. (2000) 7:1–15. 10.1080/135062800394658

[14] Simons DJ Levin DT. Change blindness. Trends Cogn Sci. (1997) 1:261–7. 10.1016/S1364-6613(97)01080-2 21223921

[15] Auvray M Gallace A Tan HZ Spence C. Crossmodal change blindness between vision and touch. Acta Psychol. (2007) 126, 79–97. 10.1016/j.actpsy.2006.10.005 17187750

[16] Rensink RA. Seeing, sensing, and scrutinizing. Vision Res. (2000) 40:1469–87. 10.1016/S0042-6989(00)00003-1 10788653

[17] Rensink RA. Internal vs. external information in visual perception. In: Proceedings of the 2nd International Symposium on Smart Graphics. Hawthorne, NY (2002). p. 63–70. 10.1145/569005.569015

[18] Henderson JM. Human gaze control during real-world scene perception. Trends Cogn Sci. (2003) 7:498–504. 10.1016/j.tics.2003.09.006 14585447

[19] Hollingworth A Henderson JM. Semantic informativeness mediates the detection of changes in natural scenes. Visual Cogn. (2000) 7:213–35. 10.1080/135062800394775

[20] LaPointe MRP Milliken B. Conflicting effects of context in change detection and visual search: a dual process account. Canad J Exp Psychol. (2017) 71:40–51. 10.1037/cep0000105 27936836

[21] Lyyra P Astikainen P Hietanen JK. Look at them and they will notice you: distractor-independent attentional capture by direct gaze in change blindness. Vis cogn. (2017) 26:25–36. 10.1080/13506285.2017.1370052

[22] Yao R Street W Simons DJ Jensen MS Street WN. Change blindness and inattentional blindness. WIREs Cognitive Science. (2011) 2:529–46. 10.1002/wcs.130 26302304

[23] Martinussen R Hayden J Hogg-Johnson S Tannock R. A meta-analysis of working memory impairments in children with attention-deficit/hyperactivity disorder. J Am Acad Child Adolesc Psychiatry. (2005) 44:377–84. 10.1097/01.chi.0000153228.72591.73 15782085

[24] Cohen AL. Performance on the Flicker Task and Conners' CPT in children with ADHD (Doctoral dissertation), Auburn University; ProQuest Dissertations and These Global, Auburn, AL (2009).

[25] Maccari L Casagrande M Martella D Anolfo M Rosa C Fuentes LJ. Change blindness in children with ADHD: A selective impairment in visual search? J Atten Disord. (2013) 17:620–7. 10.1177/1087054711433294 22334620

[26] Cohen AL Shapiro SK. Exploring the performance differences on the flicker task and the conners' continuous performance test in adults with ADHD. J Atten Disord. (2007) 11:49–63. 10.1177/1087054706292162 17606772

[27] Türkan BN Amado S Ercan ES Perçinel I. Comparison of change detection performance and visual search patterns among children with/without ADHD: evidence from eye movements. Res Dev Disabil. (2016) 49:205–15. 10.1016/j.ridd.2015.12.002 26707929

[28] Karatekin C Asarnow RF. Exploratory eye movements to pictures in childhood-onset schizophrenia and attention-deficit/hyperactivity disorder (ADHD). J Abnorm Child Psychol. (1999) 27:35–49. 10197405 10.1023/a:1022662323823

[29] Navarro O Gonzalez AL Molina AI. Experience of use of eye tracking technology with children who have attention problems. In: 2018 International Symposium on Computers in Education. Jerez (2018). 10.1109/SIIE.2018.8586721

[30] Mohammadhasani N Nucita A Iannizzotto G. Atypical visual scan path affects remembering in ADHD. J Int Neuropsychol Soc. (2019) 26:557–66. 10.1017/S135561771900136X 31826774

[31] Faul F Erdfelder E Lang AG Buchner A. G^*^ Power 3: A flexible statistical power analysis program for the social, behavioral, and biomedical sciences. Behav Res Methods. (2007) 39:175–91. 10.3758/BF03193146 17695343

[32] Hochhauser M Aran A Grynszpan O. How adolescents with autism spectrum disorder (ASD) spontaneously attend to real-world scenes: use of a change blindness paradigm. J Autism Dev Disord. (2018) 48:502–10. 10.1007/s10803-017-3343-6 29076035

[33] O'Regan KJ Deubel H Clark JJ Rensink RA. Picture changes during blinks: looking without seeing and seeing without looking. Vis Cognit. (2000) 7:191–211. 10.1080/13506280039476617931887

[34] Lavie N Tsal Y. Perceptual load as a major determinant of the locus of selection in visual attention. Percept Psychophys. (1994) 56:183–97. 10.3758/BF03213897 7971119

[35] Lavie N. Distracted and confused? Selective attention under load. Trends Cogn Sci. (2005) 9:75–82. 10.1016/j.tics.2004.12.004 15668100

[36] Prabhakar G Mukhopadhyay A Murthy L Madan M Sachin D Biswas P. Cognitive load estimation using ocular parameters in automotive. Transp Eng. (2020) 2:100008. 10.1016/j.treng.2020.100008

[37] Walter K Bex P. Cognitive load influences oculomotor behavior in natural scenes. Sci Rep. (2021) 11:1–12. 10.1038/s41598-021-91845-5 34117336 PMC8196072

[38] Forster S Robertson DJ Jennings A Asherson P Lavie N. Plugging the attention deficit: perceptual load counters Increased distraction in ADHD. Neuropsychology. (2014) 28:91–7. 10.1037/neu0000020 24219607 PMC3906797

[39] Willcutt EG Doyle AE Nigg JT Faraone SV Pennington BF. Validity of the executive function theory of attention-deficit/ hyperactivity disorder: a meta-analytic review. Biol Psychiatry. (2005) 57:1336–46. 10.1016/j.biopsych.2005.02.006 15950006

[40] Yeh YC Tsai JL Hsu WC Lin CF. A model of how working memory capacity influences insight problem solving in situations with multiple visual representations: an eye tracking analysis. Think Skills Creat. (2014) 13:153–67. 10.1016/j.tsc.2014.04.003

[41] Burgess GC Depue BE Ruzic L Willcutt EG Du YP Banich MT. Attentional control activation relates to working memory in attention-deficit/hyperactivity disorder. Biol Psychiatry. (2010) 67:632–40. 10.1016/j.biopsych.2009.10.036 20060961 PMC2953472

[42] Jayawardena G Michalek A Jayarathna S. Eye gaze metrics and analysis of AOI for indexing working memory towards predicting ADHD. arXiv [Preprint] arXiv:1906.07183 (2019).

[43] Krejtz K Duchowski A Szmidt T Krejtz I González Perilli F Pires A. Gaze transition entropy. ACM Trans Appl Percept. (2015) 13:1–20. 10.1145/2834121

[44] Munoz DP Armstrong IT Hampton KA Moore KD. Altered control of visual fixation and saccadic eye movements in attention-deficit hyperactivity disorder. J Neurophysiol. (2003) 90:503–14. 10.1152/jn.00192.2003 12672781

[45] Levin DT Simons DJ. Failure to detect changes to attended objects in motion pictures. Psychon Bull Rev. (1997) 4:501–6. 10.3758/BF03214339

